# Correlation of vascular endothelial growth factor expression with fibroblast growth factor-8 expression and clinico-pathologic parameters in human prostate cancer

**DOI:** 10.1054/bjoc.2001.1971

**Published:** 2001-08

**Authors:** A F West, M O'Donnell, R G Charlton, D E Neal, H Y Leung

**Affiliations:** Prostate Research Group, Department of Surgery, The Medical School, University of Newcastle, Newcastle upon Tyne, NE2 4HH; Department of Pathology, Freeman Hospital, Freeman Road, Newcastle upon Tyne, NE7 7DN, UK

**Keywords:** prostate cancer, benign prostatic hyperplasia (BPH), vascular endothelial growth factor (VEGF), fibroblast growth factor-8 (FGF-8), immunohistochemistry, survival

## Abstract

Vascular endothelial growth factor (VEGF) mediates neo-angiogenesis during tumour progression and is known to cooperate with the fibroblast growth factor (FGF) system to facilitate angiogenesis in a synergistic manner. In view of this, we have investigated VEGF expression in 67 cases of prostate cancer previously characterized for fibroblast growth factor-8 (FGF-8) expression. Cytoplasmic VEGF staining was detected in malignant cells in 45 out of 67 cases. Cytoplasmic staining was found in adjacent stromal cells in 32 cases, being particularly strong around nests of invasive tumour. Positive VEGF immunoreactivity in benign glands was restricted to basal epithelium. A significant association was observed between tumour VEGF and FGF-8 expression (*P* = 0.004). We identified increased VEGF immunoreactivity in both malignant epithelium and adjacent stroma and both were found to be significantly associated with high tumour stage (*P* = 0.0047 and *P* = 0.0002, respectively). VEGF expression also correlated with increased serum PSA levels (*P* = 0.01). Among positively stained tumours, VEGF expression showed a significant association with Gleason score (*P* = 0.04). Cases showing positive VEGF immunoreactivity in the stroma had a significantly reduced survival rate compared to those with negative staining (*P* = 0.037). Cases with tumours expressing both FGF-8 in the malignant epithelium and VEGF in the adjacent stroma had a significantly worse survival rate than those with tumours negative for both, or only expressing one of the two growth factors (*P* = 0.029). Cox multivariate regression analysis of survival demonstrated that stromal VEGF and tumour stage were the most significant independent predictors of survival. In conclusion, we report for the first time a correlation of both tumour and stromal VEGF expression in prostate cancer with clinical parameters as well as its correlation to FGF-8 expression. © 2001 Cancer Research Campaign http://www.bjcancer.com


*British Journal of Cancer* (2001) **85**(4), 576–583
© 2001 Cancer Research Campaign
doi: 10.1054/ bjoc.2001.1971, available online at http://www.idealibrary.com on IDEAL
http://www.bjcancer.com

### Correlation of vascular endothelial growth factor expression with fibroblast growth factor-8 expression and clinico-pathologic parameters in human prostate cancer

**AF West[1], M O'Donnell[2], RG Charlton[2], DE Neal[1] and HY Leung[1]**

[1]Prostate Research Group, Department of Surgery, The Medical School, University of Newcastle, Newcastle upon Tyne NE2 4HH; [2]Department of Pathology, Freeman Hospital, Freeman Road, Newcastle upon Tyne NE7 7DN, UK

**Summary** Vascular endothelial growth factor (VEGF) mediates neo-angiogenesis during tumour progression and is known to cooperate with the fibroblast growth factor (FGF) system to facilitate angiogenesis in a synergistic manner. In view of this, we have investigated VEGF expression in 67 cases of prostate cancer previously characterized for fibroblast growth factor-8 (FGF-8) expression. Cytoplasmic VEGF staining was detected in malignant cells in 45 out of 67 cases. Cytoplasmic staining was found in adjacent stromal cells in 32 cases, being particularly strong around nests of invasive tumour. Positive VEGF immunoreactivity in benign glands was restricted to basal epithelium. A significant association was observed between tumour VEGF and FGF-8 expression ($P$ = 0.004). We identified increased VEGF immunoreactivity in both malignant epithelium and adjacent stroma and both were found to be significantly associated with high tumour stage ($P$ = 0.0047 and $P$ = 0.0002, respectively). VEGF expression also correlated with increased serum PSA levels ($P$ = 0.01). Among positively stained tumours, VEGF expression showed a significant association with Gleason score ($P$ = 0.04). Cases showing positive VEGF immunoreactivity in the stroma had a significantly reduced survival rate compared to those with negative staining ($P$ = 0.037). Cases with tumours expressing both FGF-8 in the malignant epithelium and VEGF in the adjacent stroma had a significantly worse survival rate than those with tumours negative for both, or only expressing one of the two growth factors ($P$ = 0.029). Cox multivariate regression analysis of survival demonstrated that stromal VEGF and tumour stage were the most significant independent predictors of survival. In conclusion, we report for the first time a correlation of both tumour and stromal VEGF expression in prostate cancer with clinical parameters as well as its correlation to FGF-8 expression. © 2001 Cancer Research Campaign http://www.bjcancer.com

**Keywords**: prostate cancer; benign prostatic hyperplasia (BPH); vascular endothelial growth factor (VEGF); fibroblast growth factor-8 (FGF-8); immunohistochemistry; survival

Prostate cancer is the most common cancer in adult males in the Western world. Currently in the UK, a significant proportion of men presenting clinically with prostate cancer have advanced disease and their treatment remains unsatisfactory. Endocrine therapy (with either surgical orchiectomy or chemical androgen ablation) in men with metastatic disease aims for palliation and gives transient control of the disease for an average of 2 years (Klein, 1979; Scott et al, 1980). Prostate cancer responds poorly to combination chemotherapy and novel treatment modalities are urgently needed.

For solid tumours such as prostate cancer to develop and progress, neo-angiogenesis is critical once the tumour volume reaches 2–3 $mm^3$ (Folkman, 1990). Angiogenesis is tightly controlled by interaction between angiogenic and anti-angiogenic factors (Bouck et al, 1996). A large number of angiogenic molecules have now been identified including fibroblast growth factor-2 (FGF-2 or bFGF) and vascular endothelial growth factor (VEGF).

*Received 8 November 2000*
*Revised 25 April 2001*
*Accepted 30 April 2001*

*Correspondence to:* HY Leung

Many peptide growth factors play important roles in the pathobiology of prostate cancer. They act as mitogens, facilitating proliferation, invasion and preventing apoptosis. They mediate bi-directional interactions between stroma and epithelium as paracrine agents, further contributing to carcinogenesis and tumour progression. Peptide growth factors such as insulin-like growth factors, epidermal growth factors and members of the FGF families, and their high affinity receptor tyrosine kinases, are implicated in mediating cellular proliferation in human prostate cancer (Byrne et al, 1996; Tennant et al, 1996; Dorkin et al, 1999a).

VEGF is a soluble, dimeric 45 kDa protein that induces vascular endothelial cell proliferation and vessel hyperpermeability (Senger et al, 1983; Connolly et al, 1989; Ferrara et al, 1992). Several tumour cell types secrete it and its high affinity tyrosine kinase receptors, namely fms-like tyrosine kinase 1 (FLT-1/VEGF receptor 1) and fetal liver kinase (FLK/KDR/VEGF receptor 2), are found on vascular endothelial cells (Terman et al, 1992; deVries et al, 1992; Quinn et al, 1993) and tumour cells (Boocock et al, 1995; Ferrer et al, 1999). Positive associations have been demonstrated between tumour VEGF expression and tumour aggressiveness in several cancers including colon (Takahashi et al, 1995), breast (Toi et al, 1995) and gastric (Maeda et al, 1996) cancers.

In the prostate, expression of VEGF mRNA and protein has been reported in the malignant epithelium as well as tumour-associated stroma and a proportion of non-malignant epithelial cells (Jackson et al, 1997). Weidner et al (1993) and other groups have demonstrated a correlation between tumour angiogenesis and metastatic disease in invasive prostate cancer. In a rat prostate tumour model, increased VEGF expression was associated with enhanced microvessel density and metastatic capacity (Haggstrom et al, 2000).

Previous in vitro and in vivo work has also suggested that the VEGF and FGF systems, acting through distinct receptor kinases, may function in a synergistic manner to enhance angiogenesis and tumourigenicity (Pepper et al, 1992; Asahara et al, 1995). Furthermore, FGF-2 may also regulate VEGF expression in vascular endothelial cells via autocrine and paracrine mechanisms (Seghezzi et al, 1998). FGF-8, originally identified as the androgen-induced growth factor (Tanaka et al, 1992), is another key member of the FGF family involved in tumorigenesis. In humans, the protein exists as four isoforms, denoted FGF-8a, 8b, 8e and 8f (Gemel et al, 1996). It is widely expressed during embryogenesis (Heikinheimo et al, 1994), with only low levels of expression in some normal adult tissues such as testis, prostate and the brain (Tanaka et al, 1998; Dorkin et al, 1999a). We have previously characterized the expression of members of the FGF family in prostate cancer and identified FGF-8 to be most important (Dorkin et al, 1999b). The b isoform was overexpressed in malignant prostate epithelium; this overexpression correlated with advanced tumour stage and higher Gleason scores, and persisted in androgen-independent disease (Dorkin et al, 1999a). Here we report our study on VEGF immunoreactivity in resected prostate cancer and describe the correlation between VEGF expression and clinical parameters, and with the expression of FGF-8.

### MATERIALS AND METHODS

### Patient materials

A cohort of 67 cases of prostate cancer, diagnosed between 1989 and 1994 and previously characterized for FGF-1, -2 and -8 expression (Dorkin et al, 1999a, 1999b), were included in this study. The ages studied ranged from 49–90 years (mean 68 years) at the time of surgery. The clinical details are outlined in Table 1. A total of 23 patients were confirmed to have bony metastatic disease on isotopic bone scans, which were performed in 45 patients in this series. Archival, formalin-fixed, paraffin-embedded specimens obtained from transurethral resection of the prostate (TURP) were examined. No men had received androgen deprivation therapy prior to prostate resection. The overall median survival rate of the patients studied was 38.5 months. A total of 20 cases of benign prostatic hyperplasia (BPH), also obtained from TURP, were included in the study. Archival blocks of endometrium in early proliferative and late secretory phases of the menstrual cycle were included as positive controls for VEGF expression (Zhang et al, 1998).

### Immunohistochemistry

Initial assessment of a mouse monoclonal anti-human VEGF antibody M293 (R&D Systems, Abingdon, UK) using a variety of pre-treatment conditions, including microwave, pressure cooker, trypsin as well as no pre-treatment, failed to provide an optimal signal. A second anti-VEGF antibody was tested: the goat polyclonal antibody (AB-293-NA, R&D Systems) provided clean and reproducible signals in our hands without the need for high temperature antigen retrieval, allowing better tissue morphology. This antibody has also been preferred for similar reasons in another immunohistochemistry study (Kumar-Singh et al, 1999).

We cut 5 μm sections from tumour blocks; then they were mounted onto APES-coated slides, air-dried at 60°C, de-waxed in xylene, and then sequentially rehydrated in 100%, 70% and 50% ethanol. Endogenous peroxidase activity was blocked by incubating sections for 10 min in 9% hydrogen peroxide in distilled water. Sections were washed in running water for 5 min, and then incubated in 0.1% trypsin (ICN, Basingstoke, UK) in a 0.1% calcium chloride solution (BDH, c/o NELS, Co. Durham, UK) at 37°C (pH 7.6) for 18 min. After a further wash in running water for 5 min followed by phosphate buffered saline (PBS) for 2 min, the sections were blocked for 10 min in normal rabbit serum (GibcoBRL, Paisley, UK) diluted 1:5 in PBS and then washed in two changes of PBS over 10 min. The primary antibody was a goat polyclonal anti-human VEGF antibody (R&D systems, Abingdon, UK), diluted in normal rabbit serum to a 10 mg/ml stock. Sections were incubated in primary antibody at 4°C overnight. Pre-immune serum was applied as an internal negative control. The slides were then incubated for 30 min in biotinylated rabbit anti-goat antibody (Manarini Diagnostics, Wokingham, UK) diluted 1:60 in PBS at room temperature for 40 min, followed by 2 washes in PBS over 10 min in total. The sections were then incubated in a streptavidin HRP label (Biomen Supersensitive goat kit) diluted 1:60 in PBS for 30 min at room temperature, followed by 2 washes in

**Table 1** Clinical details

| **Stage*** | **Total number (*n* = 65)** | **Median serum PSA[†]** | **Range** | **No. with PSA data (*n* = 35)** |
|---|---|---|---|---|
| T1 | 16 | 14.9 | (1.2–65) | 4 |
| T2 | 10 | 79.5 | (21.4–98.7) | 8 |
| T3 | 20 | 30.6 | (3–5047) | 14 |
| T4 | 19 | 97.3 | (3.6–600) | 9 |
| **Gleason score** | ***n* = 67** | | | |
| 2–6 | 9 | 7.8 | (1.2–65) | 4 |
| 7 | 24 | 22.3 | (3.6–446) | 11 |
| 8–10 | 34 | 82.9 | (3–5047) | 20 |

*T-stage data unavailable for two cases; [†]Serum PSA at diagnosis – data not available for 32 cases. PSA values in nanogram ml/ (ng/ml) with range in brackets.

© 2001 Cancer Research Campaign 

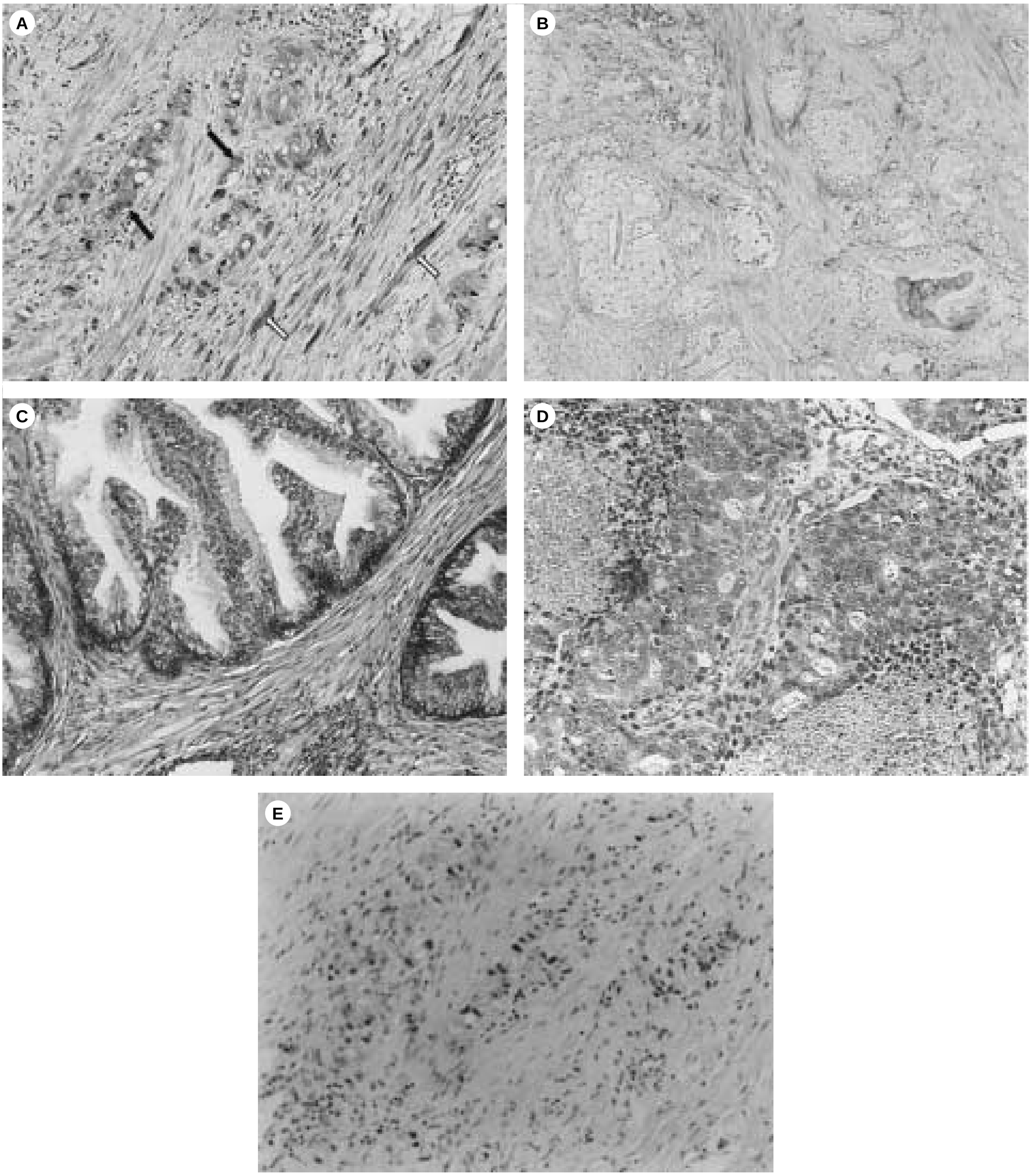

**Figure 1** Immunostaining for VEGF expression. (**A**) Gleason 8, T3M1 prostate cancer with moderate VEGF immunostaining of both tumour epithelium (black arrows) and adjacent stroma (white arrows). (**B**) Gleason 7, T4 prostate cancer with negative VEGF expression in tumour epithelium and positive expression in the peritumoural stroma. (**C**) VEGF expression in the basal cells of benign glandular epithelium. (**D**) Section of prostate cancer stained for the neuroendocrine marker, protein gene product (PGP), showing positivity for NE-type cells. (**E**) Negative control (no primary antibody) for Figure 1 (**A**) (all at magnification × 200)

PBS for 10 min each. Finally, sections were incubated in DAB (3′3′-diaminobenzidinetetrahydrochloride), counterstained with Carazzis haematoxylin, dehydrated, cleared and mounted. They were then viewed using a light microscope.

Expression of FGF-8 had previously been characterized at transcript level by in situ hybridization (ISH) (Dorkin et al, 1999a). Our current pilot data on FGF-8 immunostaining is consistent with the data from ISH. For the current study, contiguous sections from

 *© 2001 Cancer Research Campaign*

the same tissue blocks were used for each case examined for VEGF protein expression.

### Scoring of sections

Two independent observers (AFW and MO'D, a consultant histopathologist) scored all sections, with no prior knowledge of clinical data on any of the cases. An agreed scoring system for intensity of VEGF immunostaining was established by initial examination and grading of the first 20 cases. The cell type expressing VEGF was noted and the intensity of VEGF immunostaining was scored semiquantitatively as negative (–), weak (+), moderate (++) or strong (+++). A section was taken as positive when over 25% of the tumour area stained positively. Stromal VEGF expression was similarly scored, with sections taken as positive when at least 25% of the stromal area stained positively. To analyze the relationships between VEGF expression and the various clinico-pathologic features, Fisher's exact probability test was used. Trends were analyzed using the chi-square test. For comparison of serum PSA values between groups separated on the basis of intensity of staining for VEGF, the Mann–Whitney *U*-test was used. To analyze the relationship between VEGF expression and survival, the Kaplan–Meier method was used with differences in survival between groups examined using the log rank test. Multivariate Cox regression analysis was used to assess the independent predictive value of VEGF in relation to other clinico-pathologic parameters. *P*-values of less than 0.05 were considered statistically significant.

### RESULTS

### Immunohistochemical expression of VEGF in prostate cancer and BPH

Positive VEGF staining was observed in malignant prostatic epithelium in 45 out of 67 sections (67%) (Figure 1A). The staining pattern was focal and cytoplasmic, with areas of strong or moderate staining glands showing heterogeneous immunoreactivity and interspersed with areas of weak or negative staining glands. Just over half (24/45; 53%) of these cases showed predominantly moderate or strong positive staining.

VEGF immunostaining was also present in the stroma in 32 (47.8%) of the 67 cases, and was noted to be particularly strong adjacent to nests of invasive tumour, being weak or negative elsewhere. Stromal staining was observed predominantly in fibroblasts and such peri-tumoural localization appeared not to be related to the intensity of tumour epithelial immunoreactivity for VEGF (Figure 1A and B). Vessels were observed in all sections but the pattern of distribution was not specifically looked at. Vascular endothelial cells provided an internal positive control for comparison. There was a significant association between VEGF and FGF-8 expression within tumour cells ($P$ = 0.004), although FGF-8 expression in tumour epithelium did not correlate with VEGF expression in the adjacent stroma (Table 2).

In BPH ($n$ = 20), there was prominent staining of the basal cells of benign glands with weak or negative staining of the luminal cells and stroma (Figure 1C). Similarly, in cancer sections, adjacent benign glands showed VEGF immunoreactivity in the basal epithelium.

Increased VEGF in both malignant epithelium and adjacent stroma was significantly associated with high tumour stage ($P$ = 0.0047 and $P$ = 0.0002, respectively; Table 3). A significant correlation was also observed between high levels of VEGF immunoreactivity and increasing serum prostate-specific antigen (PSA) levels ($P$ = 0.01) (Table 4). There was a weak association between VEGF expression and Gleason score among the cases positive for VEGF in tumour cells (Table 5; $P$ = 0.04).

In this series, 5 men had tumours with neuroendocrine-like (NE-like) appearances and stained positively for protein gene product

**Table 2** Association between tumour FGF-8 expression and VEGF immunoreactivity in tumour and stroma

| | Tumour VEGF | | Stromal VEGF | |
|---|---|---|---|---|
| | –/+ | ++/+++ | – /+ | ++/+++ |
| FGF-8 –/+ | 21 | 4 | 12 | 9 |
| FGF-8 ++/+++ | 19 | 21 | 18 | 18 |
| | $P$ = 0.004 | | $P$ = 0.78 | |

–/+: negative/weak; ++/+++: moderate/strong. FGF-8 expression data unavailable in 2 cases; stromal VEGF expression data unavailable in 10 cases.

**Table 3** Association of VEGF expression with tumour stage

| | Tumour VEGF | | Stromal VEGF | |
|---|---|---|---|---|
| | –/+ | ++/+++ | – /+ | ++/+++ |
| Clinical stage | | | | |
| T1/2 | 22 (84.6%) | 4 (15.4%) | 18 (85.7%) | 3 (14.3%) |
| T3/4 | 20 (50%) | 20 (50%) | 12 (33.3%) | 24 (66.6%) |
| | $P$ = 0.0047 | | $P$ = 0.0002 | |
| Total | $n$ = 66* | | $n$ = 57† | |

*T-stage data unavailable in one case; †Stromal VEGF expression data unavailable for 10 cases. Intensity of VEGF signal –/+: negative or weak; ++/+++: moderate or strong.

**Table 4** Association of VEGF expression with serum PSA

| | Tumour VEGF | |
|---|---|---|
| | –/+ | ++/+++ |
| Median serum PSA* | 26.2 | 82.5 |
| Range | 1.2–600 | 7.5–5047 |
| | n = 21 | n = 14 |

$P$ = 0.01; Mann–Whitney *U*-test; –/+: negative or weak; ++/+++: moderate/strong. Serum PSA in ng/ml.

**Table 5** Association of tumour epithelial VEGF expression and Gleason score

| | Tumour VEGF | | |
|---|---|---|---|
| | – | + | ++/+++ |
| Gleason score ($n$ = 67) | | | |
| 6 and less | 5 | 3 | 1 |
| 7 | 8 | 8 | 8 |
| 8–10 | 7 | 12 | 15 |

Intensity of VEGF signal –: negative; +: weak; ++/+++: moderate/strong. $P$ = 0.04 (chi-square test for trend).

© 2001 Cancer Research Campaign

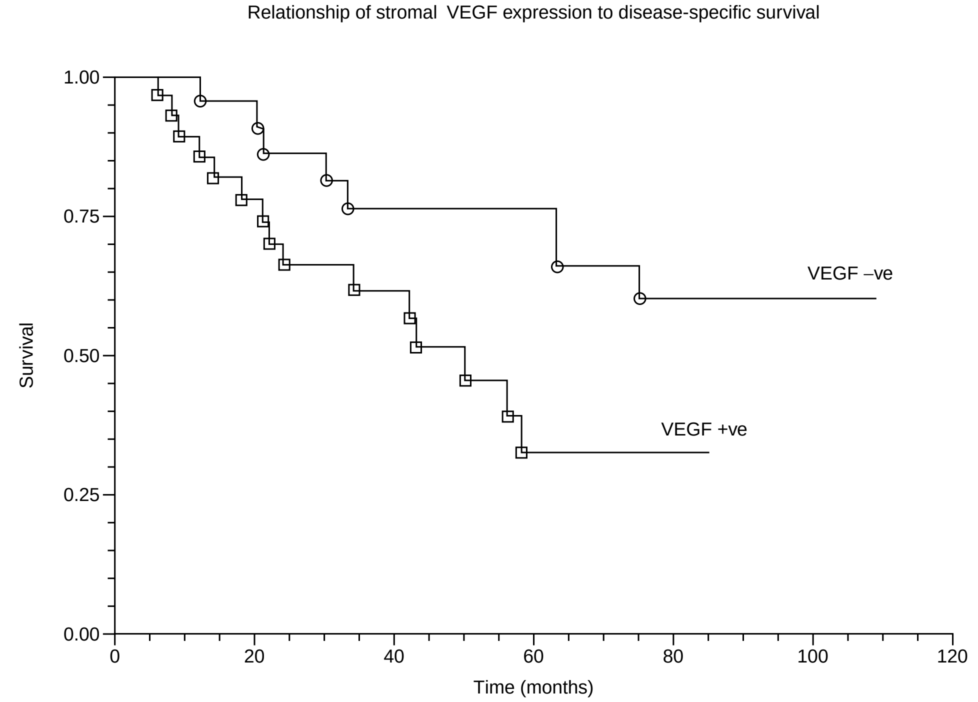

**Figure 2** Kaplan–Meier survival curves of 57 patients with prostatic carcinoma grouped according to the presence or absence of VEGF expression in the peritumoural stroma. A significant difference in disease-specific survival was found between the VEGF + group ($n$ = 32) and the VEGF – ($n$ = 25) group ($P$ = 0.037, log rank test)

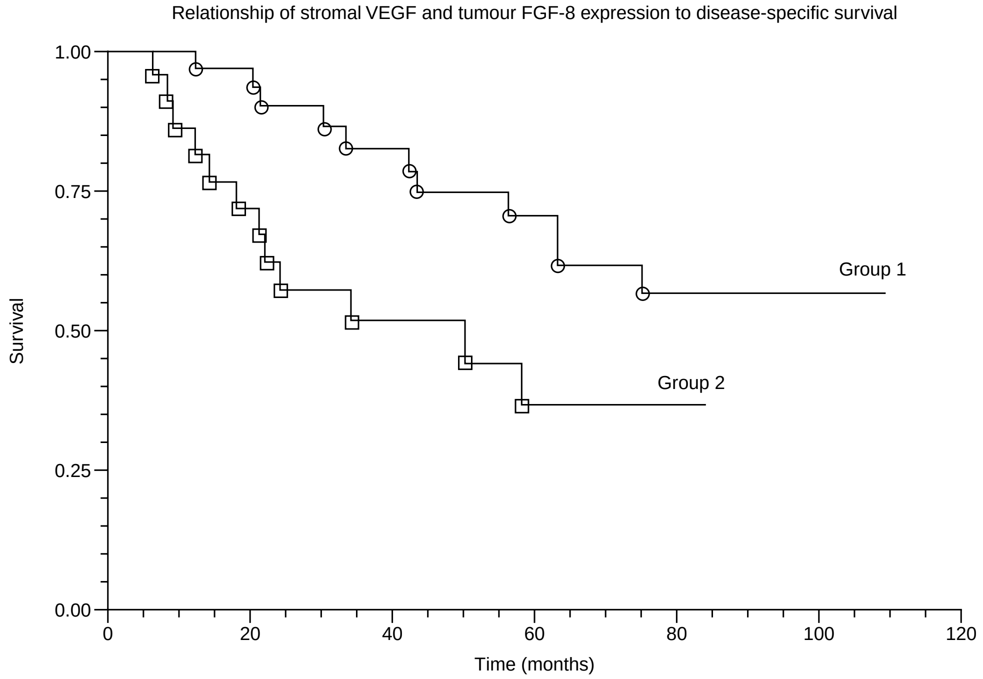

**Figure 3** Kaplan–Meier survival curves of 54 patients with prostatic carcinoma grouped according to VEGF expression in peritumoural stroma and FGF-8 expression in tumour epithelium. Group 1 ($n$ = 32): Either FGF8 +/VEGF– or FGF8–/VEGF+ or FGF8–/VEGF–; Group 2 ($n$ = 22): FGF8+/VEGF+. A significant difference in disease-specific survival was found between the two groups ($P$ = 0.029, log rank test)

(PGP) and chromogranin A, both markers for neuroendocrine cell type (Figure 1D). All five men had high-grade and high-stage disease and died from prostate cancer during follow up. The VEGF immunoreactivity in this subgroup was cytoplasmic and heterogeneous, at moderate or strong intensity.

The level of tumoural and stromal VEGF expression did not show significant associations with the presence of hot spots on isotopic bone scans. Of the total number of patients who had died from prostate cancer at the time of the study ($n$ = 22; 32.8%), with a median follow up of 27 months (range 6–109 months), 15

*© 2001 Cancer Research Campaign*

**Table 6** Cox multivariate regression analysis

| Variable | Wald chi-square | *P*-value |
|---|---|---|
| Gleason score (2–7 vs 8–10) | 0.313 | 0.576 |
| Tumour stage (T1/2 vs T3/4) | 4.1 | 0.04 |
| Tumour VEGF (–/+ vs ++/+++) | 0.702 | 0.402 |
| Stromal VEGF (– vs +) | 3.85 | 0.049 |

(62.5%) showed moderate or strong immunoreactivity for VEGF within the peri-tumoural stroma. There was no significant age difference between cases negative for VEGF staining and those that showed positive staining ($P = 0.22$). Cases showing such positive immunoreactivity for VEGF in the peri-tumoural stroma appeared to have significantly reduced disease-specific survival rates compared to those cases with tumours that were negative for stromal VEGF immunoreactivity ($P = 0.037$; Figure 2). Tumours expressing both FGF-8 in the malignant epithelium and VEGF in the adjacent stroma had a significantly worse survival rates than those cases with tumours negative for both or only expressing one of the two growth factors (Figure 3; $P = 0.029$). Although a similar relationship was also observed between tumour epithelial VEGF expression, FGF-8 expression and disease-specific survival, the association was not significant ($P = 0.44$, data not shown). In a multivariate Cox regression analysis, tumour stage ($P = 0.04$) and stromal VEGF expression ($P = 0.049$) were found to be the most significant independent indicators of disease-specific survival time (Table 6).

### DISCUSSION

In this study, VEGF immunoreactivity in resected malignant prostatic epithelium occurred in the majority of specimens (45/67, 67%), in keeping with published reports (Ferrer et al, 1997; Jackson et al, 1997). Of the tumours, 5 had NE-like appearances, and all stained strongly positive for VEGF, in keeping with data from other laboratories (Harper et al, 1996; Borre et al, 2000).

We have semi-quantitatively scored VEGF immunoreactivity in the per-tumoural stromal cells. This was previously observed in a qualitative manner (Jackson et al, 1997), but to our knowledge this report is the first to describe an association of peri-tumoural stromal VEGF expression in prostate cancer with clinicopathologic parameters. Peri-tumoural stromal VEGF immunoreactivity may result from local secretion by the malignant epithelium and uptake by the stromal cells, or more likely, induction of stromal VEGF production by tumour-derived factors in a paracrine fashion, thus propagating the process of neoangiogenesis. The latter theory is supported by our observation that the levels of VEGF immunoreactivity in the malignant glands and the adjacent peri-tumoural stroma were not directly related, consistent with findings from a recent report (Mazzucchelli et al, 2000). Although VEGF expression in tumour cells did not correlate significantly with the expression in adjacent stroma, such a correlation has been described in other cancers, such as breast cancer (Lee et al, 1998), and is thought to reflect a common stimulus to VEGF induction.

We did not specifically attempt to quantitate the extent of neovascularization, although vessels were noted in all tumour sections. Microvessel density (MVD) has been shown to be increased in malignant prostate tissue (Siegal et al, 1995), to correlate with pathologic stage (Brawer et al, 1994) and to predict the development of metastatic disease (Weidner et al, 1993). We have shown a strong association between stromal VEGF immunoreactivity and tumour stage ($P = 0.0002$), supporting its potential role in the enhancement of angiogenesis. Recent studies have demonstrated a significant association between VEGF expression and MVD in prostate cancer (Borre et al, 2000; Strohmeyer et al, 2000). We did not identify a positive correlation between VEGF expression and bone scan-detected metastases. This may be explained by the presence of micro-metastases not detectable by conventional bone scans. Alternatively, tumours expressing VEGF and FGF-8, which are associated with less favourable disease survival, may behave more aggressively to result in subsequent metastatic disease.

The significant association of increased tumour epithelial VEGF immunoreactivity with higher serum PSA values is also interesting. Serum PSA is a good marker for tumour bulk in the clinical management of prostate cancer. In this respect, VEGF overexpression may indeed enhance neo-angiogenesis to increase tumour bulk with associated elevated serum PSA.

VEGF expression in benign glandular epithelium was noted both within BPH sections and within prostate cancer specimens, as demonstrated in a previous study (Jackson et al, 1997). In this study, we further localized VEGF expression in benign glandular epithelium to the basal cell compartment, where actively dividing cells renew and differentiate into the luminal prostate epithelium. Interestingly, expression of the VEGF receptor FLK-1 in BPH tissue was demonstrated to be localized specifically to this basal population of cells in a recent study (Ferrer et al, 1999). This, combined with our findings supports a potential role for VEGF as an autocrine regulator of basal cell proliferation in BPH. It is also interesting to note that FGF-8 expression in BPH is also localized to the basal epithelium (Dorkin et al, 1999a).

We found that the frequency of high VEGF expression was significantly greater in cases with higher Gleason scores than with lower scores. The correlation between increased VEGF expression in tumour epithelium and higher tumour grades in prostate cancer has been demonstrated by some investigators (Harper et al, 1996; Borre et al, 2000), but not by others (Jackson et al, 1997).

There are in vitro (Pepper et al, 1992) and in vivo (Asahara et al, 1995) data to suggest functional cooperation between VEGF and FGF-2 in the induction and maintenance of angiogenesis. Recent data suggests that FGF-8 enhanced in vitro and in vivo growth of human prostate cancer LNCaP cells, resulting in increased proliferation rate, invasion capability and tumour bulk (Song et al, 2000). When co-cultured, FGF-8 transfected LNCaP cells also potently induced proliferation of prostate stromal cells. Hence FGF-8, similar to FGF-2, may well be capable of regulating the functions of the stromal compartment including tumour angiogenesis.

Using a cohort of patients with prostate cancer that we had previously characterized for FGF-8 expression, we examined them for a potential correlation between VEGF immunoreactivity and FGF-8 expression. Tumour, but not stromal, VEGF expression was significantly associated with FGF-8 expression. This may reflect a related mode of induction for their expression, as both FGF-8 and VEGF expression have been reported to be upregulated by androgens (Tanaka et al, 1992; Joseph et al, 1997). Although other members of the FGF family are also expressed in the prostate, neither FGF-1/acidic FGF nor FGF-2/basic FGF were found to correlate to clinical parameters as closely as FGF-8 did (Dorkin et al, 1999b). Hence, we postulate that co-expression of VEGF (particularly stromal) and FGF-8 may have a synergistic effect in the progression of human prostate cancer. Indeed, such an effect is

*© 2001 Cancer Research Campaign* 

consistent with our findings on disease-specific survival, suggesting that tumours expressing both FGF-8 and VEGF had a less favourable prognosis when compared to tumours either negative or expressing only 1 of the 2 factors. Multivariate analysis also reinforces the fact that stromal VEGF expression appears to be of greater significance than tumour epithelial VEGF expression in prediction of survival.

In conclusion, increased VEGF immunoreactivity in prostate cancer is associated with high-stage disease, higher serum PSA values, FGF-8 overexpression and worse survival. Expression of VEGF in the peri-tumoural stroma appears to be particularly important, especially in relation to prognosis. These findings are consistent with the role for VEGF as a potent angiogenic agent in the development and progression of prostate cancer. VEGF expression, in this study, was examined on TURP specimens. Given the heterogeneous and multifocal nature of prostate cancer, it would be useful to examine VEGF and FGF-8 expression in radical prostatectomy specimens. Examination of the correlation between overall stromal density in tumour sections and VEGF expression patterns would also be more accurate using radical prostatectomy specimens. Information on the immunoreactivity of VEGF and other peptide growth factors may allow better prediction of progression and more appropriate treatment protocols may thus be determined for individual groups of patients.

### REFERENCES

Asahara T, Bauters C, Zheng LP, Takeshita S, Bunting S, Ferrara N, Symes JF and Isner JM (1995) Synergistic effect of vascular endothelial growth factor and basic fibroblast growth factor on angiogenesis in vivo. *Circulation* **92** (Suppl II): 365–371

Boocock CA, Charnock-Jones DS, Sharkey AM, McLaren J, Barker PJ, Wright KA, Twentyman PR and Smith SK (1995) Expression of vascular endothelial growth factor and its receptors flt and KDR in ovarian cancer. *J Natl Cancer Inst* **87**: 506–516

Borre M, Nerstrom B and Overgaard J (2000) Association between immunohistochemical expression of vascular endothelial growth factor (VEGF), VEGF-expressing neuroendocrine-differentiated tumour cells, and outcome in prostate cancer patients subjected to watchful waiting. *Clin Cancer Res* **6**: 1882–1890

Bouck N, Stellmach V and Hsu SC (1996) How tumours become angiogenic. *Adv Cancer Res* **69**: 135–174

Brawer MK, Deering RE, Brown M, Preston SD and Bigler SA (1994) Predictors of pathologic stage in prostatic carcinoma. The role of neovascularity. *Cancer* **73**: 678–687

Byrne RL, Leung H and Neal DE (1996) Peptide growth factors in the prostate as mediators of stromal epithelial interaction. *Br J Urol* **77**: 627–633

Connolly DT, Heuvelman DM, Nelson R, Olander JV, Eppley BL, Delfino JJ, Siegel NR, Leimgruber RM and Feder J (1989) Tumour vascular permeability factor stimulates endothelial cell growth and angiogenesis. *J Clin Invest* **84**: 1470

deVries C, Escobedo J, Ueno H, Houck K, Ferrara N and Williams LT (1992) The fms like tyrosine kinase, a receptor for vascular endothelial growth factor. *Science* **255**: 989–991

Dorkin TJ, Robinson MC, Marsh C, Bjartell A, Neal DE and Leung HY (1999a) FGF8 over-expression in prostate cancer is associated with decreased patient survival and persists in androgen independent disease. *Oncogene* **18**: 2755–2761

Dorkin TJ, Robinson MC, Marsh C, Neal DE and Leung HY (1996b) aFGF immunoreactivity in prostate cancer and its co-localisation with bFGF and FGF8. *J Pathol* **189**: 564–569

Ferrara N, Houck K, Jakeman L and Leung DW (1992) Molecular and biological properties of the vascular endothelial growth factor family of proteins. *Endocr Rev* **13**: 18

Ferrer FA, Miller LJ, Andrawis RI, Kurtzman SH, Albertsen PC, Laudone VP and Kreutzer DL (1997) Vascular endothelial growth factor (VEGF) expression in human prostate cancer: in situ and in vitro expression of VEGF by human prostate cancer cells. *J Urol* **157**: 2329–2333

Ferrer FA, Miller LJ, Lindquist R, Kowalczyk P, Laudone VP, Albertsen PC and Kreutzer DL (1999) Expression of vascular endothelial growth factor receptors in human prostate cancer. *Urology* **54**: 567–572

Folkman J (1990) What is the evidence that tumours are angiogenesis dependent? *J Natl Cancer Inst* **82**: 4–6

Gemel J, Gorry M, Ehrlich GD and MacArthur CA (1996) Structure and sequence of human FGF8. *Genomics* **35**: 253–257

Haggstrom S, Bergh A and Damber J (2000) Vascular endothelial growth factor content in metastasizing and nonmetastasizing Dunning prostatic adenocarcinoma. *Prostate* **45**: 42–50

Harper ME, Glynne-Jones E, Goddard L, Thurston VJ and Griffiths K (1996) Vascular endothelial growth factor (VEGF) expression in prostatic tumours and its relationship to neuroendocrine cells. *Br J Cancer* **74**: 910–916

Heikinheimo M, Lawshe A, Shackleford GM, Wilson DB and MacArthur CA (1994) Fgf-8 expression in the post-gastrulation mouse suggests roles in the development of the face, limbs and central nervous system. *Mech Dev* **48**: 129–138

Jackson MW, Bentel JM and Tilley WD (1997) Vascular endothelial growth factor (VEGF) expression in prostate cancer and benign prostatic hyperplasia. *J Urol* **157**: 2323–2328

Joseph IB, Nelson JB, Denmeade SR and Isaac JT (1997) Androgens regulate vascular endothelial growth factor content in normal and malignant prostatic tissue. *Clin Cancer Res* **3**: 2507–2511

Klein L (1979) Prostatic carcinoma. *N Engl J Med* **300**: 824–833

Kumar-Singh S, Weyler J, Martin MJH, Vermeulen PB and Van Marck E (1999) Angiogenic cytokines in mesothelioma: a study of VEGF, FGF-1 and -2, and TGFbeta expression. *J Pathol* **189**: 72–78

Lee AHS, Dublin EA, Bobrow LG and Poulsom R (1998) Invasive lobular and invasive ductal carcinoma of the breast show distinct patterns of vascular endothelial growth factor expression and angiogenesis. *J Pathol* **185**: 394–401

Maeda K, Chung Y-S, Ogawa Y, Takatsuka S, Kang SM, Ogawa M, Sawada T and Sowa M (1996) Prognostic value of vascular endothelial growth factor expression in gastric carcinoma. *Cancer* **77**: 858–863

Mazzuchelli R, Montironi R, Santinelli A, Lucarini G, Pugnaloni A and Biagini G (2000) Vascular endothelial growth factor expression and capillary architecture in high-grade PIN and prostate cancer in untreated and androgen-ablated patients. *Prostate* **45**: 72–79

Pepper MS, Ferrara N, Orci L and Montesano R (1992) Potential synergism between vascular endothelial growth factor and basic fibroblast growth factor in the induction of angiogenesis in vitro. *Biochem Biophys Res Commun* **189**: 824–831

Quinn TP, Peters KG, deVries C, Ferrara N and Williams LT (1993) Fetal liver kinase 1 is a receptor for vascular endothelial growth factor and is selectively expressed in vascular endothelium. *Proc Natl Acad Sci USA* **90**: 7533–7537

Scott WW, Menon M and Walsh PC (1980) Hormonal therapy of prostate cancer. *Cancer* **45**: 1929–1936

Seghezzi G, Patel S, Ren CJ, Gualandris A, Pintucci G, Robbins ES, Shapiro RL, Galloway AC, Rifkin DB and Mignatti P (1998) Fibroblast growth factor-2 (FGF-2) induces vascular endothelial growth factor (VEGF) expression in the endothelial cells of forming capillaries: an autocrine mechanism contributing to angiogenesis. *J Cell Biol* **141**: 1659–1673

Senger DR, Galli SJ, Peruzzi CA, Harvey VS and Dvorak HF (1983) Tumour cells secrete a vascular permeability factor that promotes accumulation of ascites fluid. *Science* **219**: 983–985

Siegal JA, Yu E and Brawer MK (1995) Topography of neovascularity in human prostate carcinoma. *Cancer* **75**: 2545–2551

Song Z, Powell WC, Kasahara N, van Bokhoven A, Miller GJ and Roy-Burman P (2000) The effect of fibroblast growth factor 8, isoform b, on the biology of prostate carcinoma cells and their interaction with stromal cells. *Cancer Res* **60**: 6730–6736

Strohmeyer D, Rossing C, Bauerfeind A, Kaufmann O, Schlechte H, Bartsch G and Loening S (2000) Vascular endothelial growth factor and its correlation with angiogenesis and p53 expression in prostate cancer. *Prostate* **45(3)**: 216–224

Takahashi Y, Kitadai Y, Bucana CD, Cleary KR and Ellis LM (1995). Expression of vascular endothelial growth factor and its receptor, KDR, correlates with vascularity, metastasis, and proliferation of human colon cancer. *Cancer Res* **55**: 3964–3968

Tanaka A, Miyamoto K, Minamino N, Takeda M, Sato B, Matsuo H and Matsumoto K (1992) Cloning and characterization of an androgen-induced growth factor essential for the androgen-independent growth of mouse mammary carcinoma cells. *Proc Natl Acad Sci USA* **89**: 8928–8932

Tanaka A, Furuya A, Yamasaki M, Hanai N, Kuriki K, Kamiakito T, Kobayashi Y, Yoshida H, Koike M and Fukayama M (1998) High frequency of fibroblast growth factor (FGF) 8 expression in clinical prostate cancers and breast tissues,

*© 2001 Cancer Research Campaign*

immunohistochemically demonstrated by a newly established neutralising monoclonal antibody against FGF 8. *Cancer Res* **58**: 2053–2056

Tennant MK, Thrasher JB, Twomey PA, Drivdahl RH, Birnbaun RS and Plymate SR (1996) Protein and messenger ribonucleic acid (mRNA) for the type 1 insulin-like growth factor (IGF) receptor is decreased and IGF-II mRNA is increased in human prostate carcinoma compared to benign prostate epithelium. *J Clin Endocrinol Metab* **81**: 3774–3782

Terman BI, Dougher-Vermazen M, Carrion ME, Dimitrov D, Armellino DC, Gospodarowicz D and Bohlen P (1992) Identification of the KDR tyrosine kinase as a receptor for vascular endothelial growth factor. *Biochem Biophys Res Commun* **187**: 1579–1586

Toi M, Inada K, Suzuki H and Tominaga T (1995) Tumour angiogenesis in breast cancer: its importance as a prognostic indicator and the association with vascular endothelial growth factor expression. *Breast Cancer Res Treat* **36**: 193–204

Weidner N, Carroll PR, Flax J, Blumenfeld W and Folkman J (1993) Tumour angiogenesis correlates with metastasis in invasive prostate cancer. *Am J Pathol* **143**: 401

Zhang L, Scott PAE, Turley H, Leek R, Lewis CE, Gatter KC, Harris AL, Mackenzie IZ, Rees MCP and Bicknell R (1998) Validation of anti-vascular endothelial growth factor (anti-VEGF) antibodies for immunohistochemical localization of VEGF in tissue sections: expression of VEGF in the human endometrium. *J Pathol* **185**: 402–408

*© 2001 Cancer Research Campaign*
